# A Method for Simultaneous Determination of 20 *Fusarium* Toxins in Cereals by High-Resolution Liquid Chromatography-Orbitrap Mass Spectrometry with a Pentafluorophenyl Column

**DOI:** 10.3390/toxins7051664

**Published:** 2015-05-14

**Authors:** Masayoshi Tamura, Naoki Mochizuki, Yasushi Nagatomi, Koichi Harayama, Akira Toriba, Kazuichi Hayakawa

**Affiliations:** 1 Research Laboratories for Food Safety Chemistry, Asahi Group Holdings, Ltd., 1-21, Midori 1-chome, Moriya-shi, Ibaraki 302-0106, Japan; E-Mails: masayoshi.tamura@asahigroup-holdings.com (M.T.); yasushi.nagatomi@asahigroup-holdings.com (Y.N.); koichi.harayama@asahigroup-holdings.com (K.H.); 2 Graduate School of Medical Sciences, Kanazawa University, Kakuma-machi, Kanazawa-shi, Ishikawa 920-1192, Japan; 3 Research & Development Center, Asahi Group Holdings, Ltd., 1-21, Midori 1-chome, Moriya-shi, Ibaraki 302-0106, Japan; 4 Institute of Medical, Pharmaceutical and Health Sciences, Kanazawa University, Kakuma-machi, Kanazawa-shi, Ishikawa 920-1192, Japan; E-Mails: toriba@p.kanazawa-u.ac.jp (A.T.); hayakawa@p.kanazawa-u.ac.jp (K.H.)

**Keywords:** *Fusarium* toxins, LC-Orbitrap MS, pentafluorophenyl column, simultaneous determination, cereals

## Abstract

A high-resolution liquid chromatography-Orbitrap mass spectrometry (LC-Orbitrap MS) method was developed for simultaneous determination of 20 *Fusarium* toxins (nivalenol, fusarenon-X, deoxynivalenol, 3-acetyl deoxynivalenol, 15-acetyl deoxynivalenol, HT-2 toxin, T-2 toxin, neosolaniol, diacetoxyscirpenol, fumonisin B_1_, fumonisin B_2_, fumonisin B_3_, fumonisin A_1_, fumonisin A_2_, fumonisin A_3_, zearalenone, α-zearalenol, β-zearalenol, α-zearalanol, and β-zearalanol) in cereals. The separation of 20 *Fusarium* toxins with good peak shapes was achieved using a pentafluorophenyl column, and Orbitrap MS was able to detect accurately from cereal matrix components within ±0.77 ppm. The samples were prepared using a QuEChERS kit for extraction and a multifunctional cartridge for purification. The linearity, repeatability, and recovery of the method were >0.9964, 0.8%–14.7%, and 71%–106%, respectively. Using this method, an analysis of 34 commercially available cereals detected the presence of deoxynivalenol, 15-acetyl deoxynivalenol, fumonisin B_1_, fumonisin B_2_, fumonisin B_3_, fumonisn A_1_, fumonisin A_2_, fumonisin A_3_, and zearalenone in corn samples with high concentration and frequency. Trichothecenes was detected from wheat samples with high frequency; in particular, the concentration of deoxynivalenol was high. Conversely, α-zearalenol, β-zearalenol, α-zearalanol, and β-zearalanol were not detected in any of the samples.

## 1. Introduction

**Figure 1:**
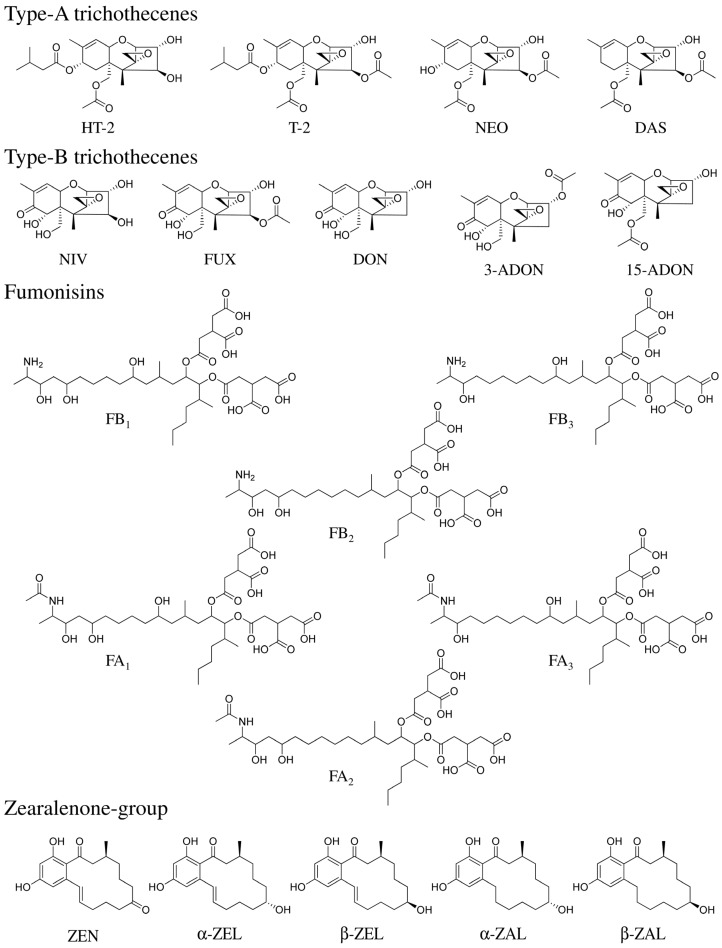
Structures of *Fusarium* toxins. Abbreviations; HT-2, HT-2 toxin; T-2, T-2 toxin; NEO, neosolaniol; DAS, diacetoxyscirpenol; NIV, nivalenol; FUX, fusarenon-X; DON, deoxynivalenol, 3-ADON, 3-acetyl deoxynivalenol; 15-ADON, 15-acetyl deoxynivalenol; FB_1_, fumonisin B_1_; FB_2_, fumonisin B_2_; FB_3_, fumonisin B_3_; FA_1_, fumonisin A_1_; FA_2_, fumonisin A_2_; FA_3_, fumonisin A_3_; ZEN, zearalenone; α-ZEL, α-zearalenol; β-ZEL, β-zearalenol; α-ZAL, α-zearalanol; and β-ZAL, β-zearalanol.

*Fusarium* is a genus of fungi that is distributed worldwide in the soil and some are known to cause “head blight” disease on cereals. These species infest growing cereals (corn, wheat, and barley), making seeds rancid thus leading to a reduction in crop quality and yield. For this reason, these *Fusarium* species have a significant economic impact. Certain *Fusarium* species are also known to produce mycotoxins [1,2,3,4]. Mycotoxins are toxic secondary metabolite compounds produced by fungi that can cause severe health problems in humans and animals. Although enniatines, moniliformin, and beauvericin, which are among the mycotoxins produced by *Fusarium* species (=*Fusarium* toxins), have attracted great attention recently in the European Union (EU) [5]; the major *Fusarium* toxins are found in the trichothecenes, fumonisins, and zearalenone-group (Figure 1). *Fusarium* toxins are frequently detected in food, often at high concentrations. Therefore, it is highly likely that human and animal health is influenced by *Fusarium* toxins. Additionally, co-contamination with several *Fusarium* toxins in cereals has been reported [4,6,7,8,9,10], and monitoring and control of *Fusarium* toxins in the food supply is necessary for food hygiene.

Trichothecenes, produced by *Fusarium culmorum*, *F. graminearum*, *F. sporotrichioides*, and *F. poae*, are known to cause diarrhea, emesis, and inflammation [1,2,3,4]. These toxins are classified into 4 types; type-A, including HT-2 toxin (HT-2), T-2 toxin (T-2), neosolaniol (NEO), and diacetoxyscirpenol (DAS) and type-B, including nivalenol (NIV), fusarenon-X (FUX), deoxynivalenol (DON), 3-acetyl deoxynivalenol (3-ADON), and 15-acetyl deoxynivalenol (15-ADON) are important for food hygiene. The 0.06 μg/kg body-weight (bw)/day for HT-2 and T-2 (alone or in combination) and 1 μg/kg bw/day for DON and its acetylated derivatives (3-ADON and 15-ADON) were set as the provisional maximum tolerable daily intake (PMTDI) by the FAO/WHO Joint Expert Committee on Food Additives (JECFA) [11,12]. Additionally, DON, HT-2, and T-2 levels in cereals are regulated in the EU and the United States (US) [13,14,15], and the maximum limit of DON in cereals and cereal products is under discussion by the CODEX Alimentarius Commission (CODEX).

Fumonisins are produced by *F. proliferatum* and *F. verticillioides*. Several analogs have been discovered, including the fumonisin A-series (FAs), fumonisin B-series (FBs), fumonisin C-series, and fumonisin P-series. Among these, FBs are well known corn contaminants. They constitute a major health risk as they may cause equine leukoencephalomalacia porcine pulmonary edema and induce esophageal cancer in human [1,2,3,4]. FBs are classified as 2B (*possibly carcinogenic to humans*) by the International Agency for Research on Cancer (IARC) [16]. The PMTDI was set at 2 μg/kg bw/day for FB_1_, FB_2_, and FB_3_ (alone or in combination) by JECFA [11]. FBs also have regulatory limits in corn in the EU and US [14,15]. The CODEX has set maximum FBs (FB_1_ and FB_2_) levels of 4 mg/kg in raw maize grain and 2 mg/kg in maize flour and maize meal [17]. We have previously reported that commercially available corns contaminated with FBs were also contaminated with FAs [18,19]. Similar to FBs, there is a toxicity report suggesting that FAs can inhibit sphingosine *N*-acyltransferase [20]. Therefore, FAs associated with *Fusarium* toxins should be monitored.

Zearalenone (ZEN), which is produced by *F. culmorum* and *F. graminearum*, has the ability to bind estrogen receptors and induces estrogenic syndromes including uterine enlargement, swelling of the vulva and mammary glands, and pseudopregnancy [1,2,3,4,21]. The PMTDI for ZEN has been set at 0.25 μg/kg bw/day by the European Food Safety Authority (EFSA) [22], and regulatory limits have also been set in the EU [14]. Additionally, α-zearalenol (α-ZEL), β-zearalenol (β-ZEL), α-zearalanol (α-ZAL), and β-zearalanol (β-ZAL) are known derivatives (reduced metabolites) of ZEN [23,24] that also have estrogenic activity. Their relative binding affinities for estrogen receptors are α-ZEL > α-ZAL > β-ZAL > ZEN > β-ZEL. α-ZAL is also used as growth promoter in livestock in the US. α-ZAL and β-ZAL have not been reported in cereals; however, *Rhizopus* species, which is a fungus that exists on cereals during storage [25], has the ability to transform ZEN to α-ZAL [26]. Therefore, there is a risk that α-ZAL and β-ZAL are present in cereals in the food supply. Based on its high estrogenic activity, not only ZEN but also its reduced metabolites should be monitored.

There are various reports of analysis method for mycotoxins in cereals and cereal products [27,28], including methods reported in recent years using simultaneous analysis by liquid chromatography (LC) with tandem quadrupole mass spectrometry (MS/MS) [9,10,23,29,30,31,32,33,34]. MS/MS enables analysis of trace amounts of mycotoxins in food with complicated matrix components because MS/MS separates ion compounds dependent on their molecular weight and other compounds using two stages of mass filters. However, co-eluting isomers are difficult to distinguish by MS/MS because they share similar structures and have the same molecular weight. *Fusarium* toxins include three pairs of regioisomers, 3-ADON/15-ADON, FB_2_/FB_3_, and FA_2_/FA_3_, and two pairs of stereoisomers, α-ZEL/β-ZEL and α-ZAL/β-ZAL, as described above. For accurate quantification, it is necessary to separate these isomers by LC. Previous analytical methods to determine the presence of *Fusarium* toxin isomers were conducted using only a few pairs of isomers in a method (e.g., a method for 3-ADON/15-ADON and FB_2_/FB_3_ [9], for FB_2_/FB_3_ and FA_2_/FA_3_ [19], for α-ZEL/β-ZEL and α-ZAL/β-ZAL [23,30], and for 3-ADON/15-ADON, FB_2_/FB_3_, and α-ZEL/β-ZEL [34]). There is currently no method for simultaneous analysis of the three groups of *Fusarium* toxins including five pairs of their isomers. Because cereals have a risk of co-contamination with different groups of *Fusarium* toxins, it is desirable to analyze all of these toxins simultaneously.

There are reports of simultaneous analysis by high-resolution mass spectrometry, including Orbitrap mass spectrometry (Orbitrap MS) and time-of-flight mass spectrometry (TOF MS) instead of MS/MS [19,35,36]. Orbitrap MS enables accurate measurement of mass up to 5 significant digits, allowing subtle differences in molecular weight to be easily distinguished. Thus, Orbitrap MS is useful for not only estimation of unknown compounds, but also for the accurate detection of known compounds. Therefore, Orbitrap MS is also adaptable for analysis of trace amounts in food with complex matrix components.

In the present study, we examined a method for the simultaneous analysis of 20 *Fusarium* toxins including isomers. The mycotoxins tested are NIV, FUX, DON, 3-ADON, 15-ADON, HT-2, T-2, NEO, DAS, FB_1_, FB_2_, FB_3_, FA_1_, FA_2_, FA_3_, ZEN, α-ZEL, β-ZEL, α-ZAL, and β-ZAL. Additionally, this simultaneous analysis method was used to detect and quantify all 20 *Fusarium* toxins in cereal samples purchased in markets.

## 2. Results and Discussion

### 2.1. Separation of 20 Fusarium Toxins Using a Pentafluorophenyl Column

**Figure 2:**
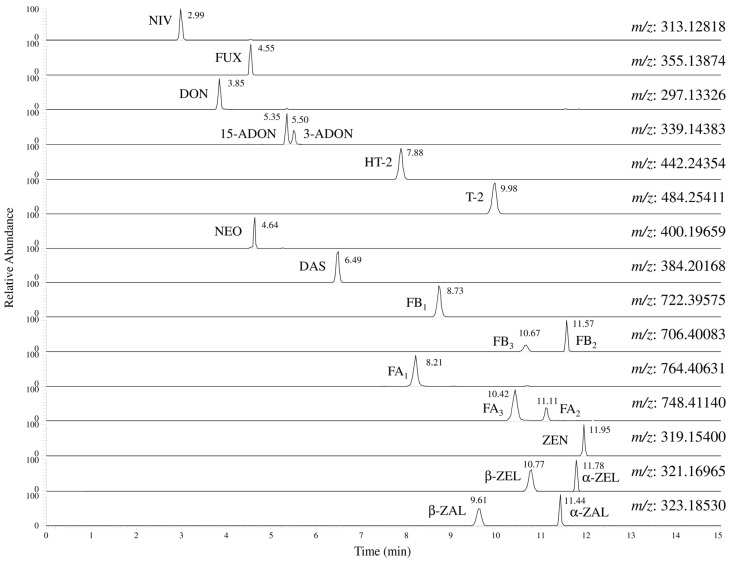
Chromatograms of the 20 *Fusarium* toxins using a Mastro C18. The analytical sample was 200 μg/L standards in neat solvent. Extraction mass window was ± 5 ppm. Abbreviations; NIV, nivalenol; FUX, fusarenon-X; DON, deoxynivalenol, 3-ADON, 3-acetyl deoxynivalenol; 15-ADON, 15-acetyl deoxynivalenol; HT-2, HT-2 toxin; T-2, T-2 toxin; NEO, neosolaniol; DAS, diacetoxyscirpenol; FB_1_, fumonisin B_1_; FB_2_, fumonisin B_2_; FB_3_, fumonisin B_3_; FA_1_, fumonisin A_1_; FA_2_, fumonisin A_2_; FA_3_, fumonisin A_3_; ZEN, zearalenone; α-ZEL, α-zearalenol; β-ZEL, β-zearalenol; α-ZAL, α-zearalanol; and β-ZAL, β-zearalanol.

Initially, LC separation was examined using an octadecylsilane (ODS) column, Mastro C18, as referenced in a previous report [10]. The separation of the 20 *Fusarium* toxins were attempted using a 10 mM aqueous solution of ammonium acetate and a 2% acetic acid solution in methanol (MeOH) as mobile phases. The chromatograms of 200 μg/L of standards in neat solvent with the ODS column are shown in Figure S1. 3-ADON and 15-ADON were not completely separated under the conditions. Thus, a pentafluorophenyl (PFP) column, Mastro PFP, was tested. The PFP column enables separation of regioisomers and stereoisomers by electrostatic interaction of fluorine atoms in functional groups [37,38]. The chromatograms of 200 μg/L of standards in neat solvent with the PFP column are shown in Figure 2. All 20 *Fusarium* toxins were completely separated with good peak shapes. It was assumed that separation of 3-ADON and 15-ADON occurred using the PFP column because of differences in the hydroxyl group position responsible for their separation activity. In this case, 3-ADON, where the hydroxyl groups are more proximate to each other, showed stronger electrostatic interaction with the PFP functional group than 15-ADON.

**Figure 3:**
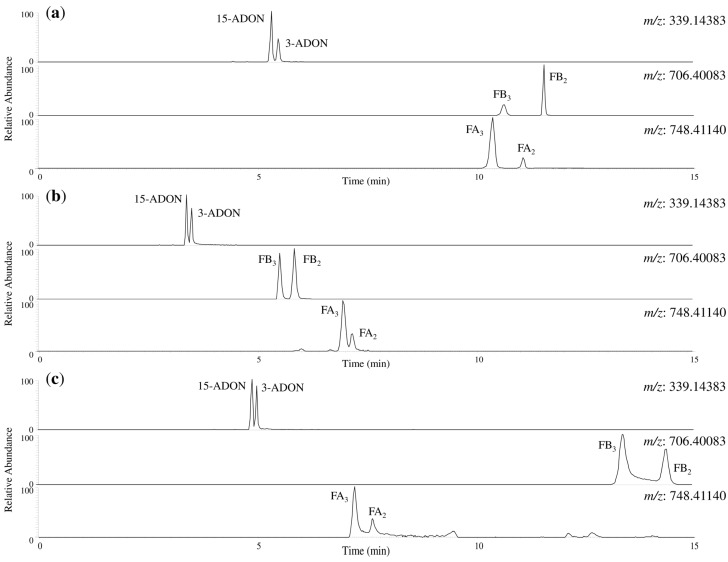
Chromatograms of 3-ADON/15-ADON, FB_2_/FB_3_, and FA_2_/FA_3_ using each PFP column. (**a**) Mastro PFP; (**b**) ACQUITY UPLC CSH Fluoro-Phenyl; (**c**) Discovery HS F5. The analytical sample was 200 μg/L standards in neat solvent. Extraction mass window was ±5 ppm. Abbreviations; 3-ADON, 3-acetyl deoxynivalenol; 15-ADON, 15-acetyl deoxynivalenol; FB_2_, fumonisin B_2_; FB_3_, fumonisin B_3_; FA_2_, fumonisin A_2_; FA_3_, fumonisin A_3_.

Additionally, peak separations of isomers were compared using ACQUITY UPLC CSH Fluoro-Phenyl and Discovery HS F5 under the same gradient conditions. Characteristic chromatograms of 3-ADON/15-ADON, FB_2_/FB_3_, and FA_2_/FA_3_ are shown in Figure 3. The separation of α-ZEL/β-ZEL and α-ZAL/β-ZAL by both PFP columns was relatively good. However, the separation of 3-ADON/15-ADON and FA_2_/FA_3_ was insufficient, and minor peak tailings were observed by ACQUITY UPLC CSH Fluoro-phenyl. Moreover, fumonisin peaks showed intense peak tailings using Discovery HS F5. Although a multi-analyte method is a compromise with no ideal conditions for all compounds, based on these results, Mastro PFP, which enabled separation of the 20 *Fusarium* toxins with good peak shapes, was the adopted method.

### 2.2. Detection of 20 Fusarium Toxins Using an LC-Orbitrap MS

**Figure 4:**
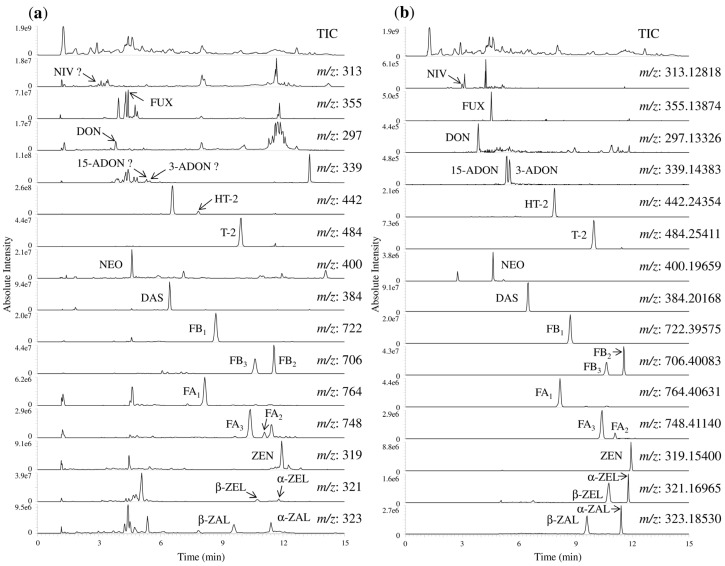
Extracted ion chromatograms of the 20 *Fusarium* toxins using nominal mass and accurate mass. (**a**) Nominal mass (extraction mass window ± 0.5 unit); (**b**) accurate mass. The analytical sample was corn spiked with 100 μg/kg standards. Abbreviations; TIC, total ion chromatogram; NIV, nivalenol; FUX, fusarenon-X; DON, deoxynivalenol, 3-ADON, 3-acetyl deoxynivalenol; 15-ADON, 15-acetyl deoxynivalenol; HT-2, HT-2 toxin; T-2, T-2 toxin; NEO, neosolaniol; DAS, diacetoxyscirpenol; FB_1_, fumonisin B_1_; FB_2_, fumonisin B_2_; FB_3_, fumonisin B_3_; FA_1_, fumonisin A_1_; FA_2_, fumonisin A_2_; FA_3_, fumonisin A_3_; ZEN, zearalenone; α-ZEL, α-zearalenol; β-ZEL, β-zearalenol; α-ZAL, α-zearalanol; and β-ZAL, β-zearalanol.

Next, the detection of known compounds in cereal matrix components was confirmed with accurate mass measurement using Orbitrap MS. The extracted ion chromatograms using accurate mass and nominal mass were compared using a prepared corn sample spiked with 100 μg/kg *Fusarium* toxin standards (Figure 4). In total ion chromatogram (TIC), contaminating compounds from all matrix components were detected within the retention time of each *Fusarium* toxin. The corn sample was prepared using the method described in Section 3.2, under “*Sample Preparation*”. In the extracted ion chromatograms using nominal mass, NIV, 3-ADON, and 15-ADON could not be distinguished from matrix components, and the peaks were buried in cereal matrix components. In contrast, the extracted ion chromatography using accurate mass showed clear peaks for all 20 *Fusarium* toxins in the corn sample. These results suggested that the accurate masses measurement was useful for detection of *Fusarium* toxins in food. 

The mass error is the difference between measured mass and theoretical mass. In particular, a smaller mass error indicates that measured mass is closer to the theoretical mass and that known compounds are able to be detected with high accuracy. The mass error was confirmed using 200 μg/L *Fusarium* toxin standards in neat solvent, a corn sample spiked with 100 μg/kg standards of *Fusarium* toxins, and a reference corn sample (MTC-9999E) that was naturally contaminated with mycotoxins (DON, HT-2, T-2, FB_1_, FB_2_, FB_3_, and ZEN). Table S1 summarizes the measured mass and mass error of the standard, the corn sample with the 20 *Fusarium* toxins, and MTC-9999E. The mass errors were within ± 0.30 ppm for the standard and within ± 0.77 ppm for the corn samples. Because a mass error within ± 5 ppm was used as the criterion for compound identification in accordance with guidelines established by the European Commission [39], we confirmed that the high-resolution measurement using Orbitrap MS enabled to detect accurately of the 20 *Fusarium* toxins in cereal matrix components.

### 2.3. Method Validation for Determination of 20 Fusarium Toxins

Extraction with a QuEChERS kit followed by purification using a Multistep 229 Ochra multifunctional cartridge was used for sample preparation. QuEChERS stands for “Quick, Easy, Cheap, Effective, Rugged, and Safe”, and it was originally developed for the determination of pesticide residues [40]. Although a MultiSep 229 Ochra multifunctional cartridge has been used for preparation of ochratoxins, which are major mycotoxins, our previous studies indicate that this cartridge is also useful for simultaneous purification of mycotoxins including NIV, DON, HT-2, T-2, afratoxin (AF) B_1_, AFB_2_, AFG_1_, AFG_2_, FB_1_, FB_2_, FB_3_, ZEN, and ochratoxin A [10,41]. Additionally, previous reports have used this preparation during quantitative analysis of NIV, DON, HT-2, T-2, ZEN, FB_1_, FB_2_, FB_3_, FA_1_, FA_2_, and FA_3_ in corn samples [10,19]; thus, it was considered to be a viable method for analysis of the other *Fusarium* toxins in this study, namely, FUX, 3-ADON, 15-ADON, NEO, DAS, α-ZEL, β-ZEL, α-ZAL, and β-ZAL. The method for the quantification of the 20 *Fusarium* toxins was evaluated using prepared corn, wheat, and barley samples spiked with *Fusarium* toxin standards. Samples in which it was confirmed beforehand that *Fusarium* toxins were not detected or were detected at very low concentrations by using the preparation method and LC-Orbitrap MS condition described in Section 3.2 and Section 3.3, under “*Sample preparation*” and “*LC-Orbitrap MS Analysis*” were selected. The results are shown in Table 1. The linearity, repeatability, and recovery were acceptable at >0.996, 0.8%–14.7%, and 71%–106%, respectively. The recovery indicates the extraction recovery using the test sample spiked *Fusarium* toxins before extraction. The definition of the limit of detection and limit of quantification is not really applicable for high-resolution mass spectrometric methods [36] because the high mass accuracy causes only limited noise and sometimes no noise to be visible. However, to ensure quantification, a certain degree of confidence is required. Therefore, the limit of quantification in the method was the lowest calibration level (=5 μg/kg). Additionally, the analytical levels of DON, HT-2, T-2, FB_1_, FB_2_, FB_3_, and ZEN in reference corn samples (DC-617, FC-443, ZC-327, MTC-9990, and MTC-9999E) were within the acceptance limits. Because individual concentrations of FB_1_ and FB_2_ in the MTC-9999E sample exceeded the range of the calibration curve, the prepared sample was diluted 10-fold by dilution solvent (10 mM aqueous ammonium acetate/acetonitlire (MeCN) (85:15, *v/v*)) prior to analysis.

A “dilute-and-shoot” approach for multi-mycotoxin analysis was reported by Sulyok *et al.* [34]. This approach is easy because it only requires extraction with a solvent (e.g., water/MeCN); thus, large amounts of matrix components are present. Although peaks can be detected from matrix components by Orbitrap MS, we consider that the sample preparation, especially the purification process, is very important for stable and consecutive quantification. Additionally, although the method is for quantification of 87 analytes including 3-ADON/15-ADON, FB_2_/FB_3_, and α-ZEL/β-ZEL; the separation of 3ADON/15ADON is insufficiently with consideration of their retention time. Moreover, because 3-ADON is detected in negative mode and 15-ADON in positive mode in this method, it needs two chromatographic run per a sample in each positive and negative mode. Therefore, 3-ADON and 15-ADON were not capable of simultaneous analysis. In comparison with the method reported by Sulyok, our method is useful for the following reasons: (1) the method was capable of simultaneous analysis at a run in only positive mode because 3-ADON and 15-ADON are separated completely; (2) the calibration curves of target toxins cover wide ranges including nearly equal recovery and repeatability; (3) the pollution of the instrument by matrix compounds is less likely due to the applied clean-up step. Thus, we have successfully developed a method for simultaneous determination of 20 *Fusarium* toxins in corn, wheat, and barley samples.

### 2.4. Quantification of the 20 Fusarium Toxins in Cereal Samples

The concentrations of the 20 *Fusarium* toxins in commercial cereal samples, which included 13 corn samples, 12 wheat samples, and 9 barley samples, were quantified using our simultaneous analysis method. The reference corn samples (DC-617, FC-443, ZC-327, MTC-9990, and MTC-9999E), which were contaminated with several *Fusarium* toxins, were selected for analysis. The concentrations were calculated based on standard addition method to compensate losses during sample preparation and due to matrix effects. Therefore, it was not necessary to separately correct the values for the recovery. The results for the detected *Fusarium* toxins are shown in Table 2 and Table 3. No value is provided if no distinct peak was detected.

In the commercial corn samples (C-1–13 in Table 2), FUX, DON, 3-ADON, 15-ADON, T-2, DAS, FB_1_, FB_2_, FB_3_, FA_1_, FA_2_, FA_3_, and ZEN were detected. DON, 15-ADON, FB_1_, FB_2_, FB_3_, FA_1_, FA_2_, FA_3_, and ZEN were detected in over half of the samples, while FB_1_, FB_2_, and FB_3_ were detected at high concentrations in all corn samples. FB_1_ has the highest concentration, with a maximum concentration of 1.30 mg/kg. Higher concentrations of DON, 15-ADON, and ZEN were detected more frequently in corn samples than in wheat and barley samples, with maximum concentrations of DON, 15-ADON, and ZEN detected at 1.11 mg/kg, 145 μg/kg, and 148 μg/kg, respectively. NIV, HT-2, NEO, α-ZEL, β-ZEL, α-ZAL, and β-ZAL were not detected. The reference corn samples had the same trend as commercial corn samples. DON, 15-ADON, FB_1_, FB_2_, FB_3_, FA_1_, FA_2_, FA_3_, and ZEN were detected in all of the samples, whereas α-ZEL, β-ZEL, α-ZAL, and β-ZAL were not detected. Interestingly, these results revealed that corn samples have a high risk of co-contamination with multiple groups of *Fusarium* toxins.

In wheat samples (W-1–12 in Table 3), trichothecenes were detected, and the NIV, FUX, DON, HT-2, and T-2 were detected in over half of the samples. Specifically, the concentration of DON was high, with a maximum concentration of 451 μg/kg. FB_1_, FB_2_, and ZEN were detected in some samples; however, the concentration was relatively low. FB_3_, FA_1_, FA_2_, FA_3_, α-ZEL, β-ZEL, α-ZAL, and β-ZAL were not detected in any of the wheat samples. These results confirmed that wheat samples were co-contaminated with trichothecenes. Although co-contamination with trichothecenes was observed in barley samples (B-1–9 in Table 3), the rate of detection and the concentrations were relatively low. The maximum concentration was observed for DON, at 116 μg/kg. α-ZEL, β-ZEL, α-ZAL, and β-ZAL were not detected in any cereal samples. Although these compounds are the derivatives (reduced metabolites) of ZEN, this finding indicates that the risk of ZEN being metabolized and reduced by the other microorganisms during cereal storage may be low.

These results confirmed that cereals have a risk of co-contamination with *Fusarium* toxins. Corn has an especially high risk of co-contamination with different groups of *Fusarium* toxins, namely, trichothecenes, fumonisins, and zearalenone-group, at high concentrations.

**Table 1:** Performance of the method.

*Fusarium* Toxins		Corn			Wheat			Barley		
		Linearity (*r*) ^(a)^	Repeatability (%) ^(b)^	Recovery (%) ^(c)^	Linearity (*r*)	Repeatability (%)	Recovery (%)	Linearity (*r*)	Repeatability (%)	Recovery (%)
Trichothecenes	NIV	0.9997	2.1	76	0.9999	5.4	71	0.9996	5.1	78
	FUX	0.9995	4.4	87	0.9998	6.2	93	0.9995	7.8	102
	DON	0.9993	4.2	80	0.9996	6.0	89	0.9968	4.6	82
	3-ADON	0.9995	5.7	92	0.9992	5.6	89	0.9978	5.8	88
	15-ADON	0.9989	2.4	95	0.9990	5.0	99	0.9982	6.8	96
	HT-2	0.9999	1.4	98	0.9998	1.0	101	0.9991	3.4	98
	T-2	0.9998	0.9	93	0.9994	1.7	95	0.9989	1.0	94
	NEO	0.9986	4.7	98	0.9987	2.9	99	0.9964	7.0	100
	DAS	0.9989	1.0	97	0.9979	1.5	97	0.9967	2.5	97
Fumonisins	FB_1_	0.9999	1.6	96	0.9992	2.7	85	0.9998	2.6	93
	FB_2_	0.9994	2.4	102	0.9985	3.8	91	0.9991	3.6	94
	FB_3_	0.9998	0.8	104	0.9998	3.4	93	0.9997	3.1	94
	FA_1_	0.9991	1.5	100	0.9997	2.3	97	0.9997	1.6	96
	FA_2_	0.9999	11.9	93	0.9999	6.7	106	0.9986	14.7	98
	FA_3_	0.9999	2.6	97	0.9996	2.6	97	0.9992	2.0	97
Zearalenone-group	ZEN	0.9998	3.4	82	0.9995	4.0	90	0.9997	7.3	84
	α-ZEL	0.9998	6.1	86	0.9994	2.4	91	0.9979	4.3	79
	β-ZEL	0.9993	3.1	95	0.9997	5.6	84	0.9988	7.6	92
	α-ZAL	0.9998	2.7	78	0.9998	6.4	97	0.9984	12.4	82
	β-ZAL	0.9990	6.8	86	0.9999	4.1	99	0.9984	6.2	78

^(a)^ Concentration range of linearity, 5–5000 μg/kg; ^(b)^ *n* = 5, The samples were spiked with 100 μg/kg; ^(c)^ *n* = 1, The samples were spiked with 100 μg/kg. Abbreviations: NIV, nivalenol; FUX, fusarenon-X; DON, deoxynivalenol, 3-ADON, 3-acetyl deoxynivalenol; 15-ADON, 15-acetyl deoxynivalenol; HT-2, HT-2 toxin; T-2, T-2 toxin; NEO, neosolaniol; DAS, diacetoxyscirpenol; FB_1_, fumonisin B_1_; FB_2_, fumonisin B_2_; FB_3_, fumonisin B_3_; FA_1_, fumonisin A_1_; FA_2_, fumonisin A_2_; FA_3_, fumonisin A_3_; ZEN, zearalenone; α-ZEL, α-zearalenol; β-ZEL, β-zearalenol; α-ZAL, α-zearalanol; and β-ZAL, β-zearalanol.

**Table 2:** Concentration of *Fusarium* toxins in corn samples.

Sample	Concentration of *Fusarium* toxins [μg/kg]															
	Trichothecenes									Fumonisins						Zearalenone-group
	NIV	FUX	DON	3-ADON	15-ADON	HT-2	T-2	NEO	DAS	FB_1_	FB_2_	FB_3_	FA_1_	FA_2_	FA_3_	ZEN
C-1	^(a)^		63.7		10.6		<5			373	70.9	54.8	34.7	53.7	5.44	<5
C-2		<5	15.3		<5				<5	229	41.4	19.0	17.6	23.9	<5	
C-3			16.0		<5					32.3	8.66	<5	<5	<5		
C-4		<5							<5	154	30.6	13.8	11.0	13.3	<5	
C-5			10.5		<5					67.3	12.5	11.5	8.29	11.0	<5	<5
C-6			53.3		14.8		<5		<5	924	171	122	87.8	168	11.6	13.7
C-7			96.1		18.0		<5			526	82.9	60.2	38.5	66.6	<5	30.2
C-8		<5	401		145		<5			38.3	<5	<5				81.7
C-9			154	<5	34.9					40.8	10.9	<5	<5	<5		19.9
C-10		8.39	135	5.57	38.1					413	45.6	60.5	34.7	47.3	7.52	6.79
C-11			214	<5	26.1		<5			1.30^c)^	291	193	106	93.9	6.76	65.8
C-12		5.62	1.11 ^(c)^	12.6	47.4					54.8	14.9	<5	<5			148
C-13										466	85.0	77.3	51.4	42.6	5.09	
DC-617	<5 ^(b)^		4.82 ^(c)^ [4.2–6.4] ^(c) (d)^		372	29.5	13.9	<5		2.48 ^(c)^	486	263	563	677	71.0	592
FC-443			99.8		16.3	<5	<5	<5		3.69 ^(c)^ [2.3–4.9] ^(c)^	786 [0.5–1.1] ^(c)^	244 [0.2–0.4] ^(c)^	522	705	74.9	<5
ZC-327			2.57 ^(c)^	27.5	241					1.56 ^(c)^	291	150	262	378	30.2	1.72 [1.08–1.85]
MTC-9990		<5	1.78 ^(c)^ [1.6–2.2] ^(c)^	10.5	153	16.2	6.73	<5	<5	1.14 ^(c)^ [1.0–1.6] ^(c)^	181 [0.15–0.25] ^(c)^	125	257	370	40.8	284
MTC-9999E	23.0	<5	2.16 ^(c)^ [2.1–3.1] ^(c)^	7.01	168	368 [350–697]	153 [142–386]	19.4	<5	28.3 ^(c)^ [20.7–35.9] ^(c)^	5.39 ^(c)^ [5.2–9.0] ^(c)^	1.36 ^(c)^ [1.2–2.2] ^(c)^	2.37	2.61	182	323 [239–465]

^(a)^ The blank cells indicate that no peak was detected; ^(^^b)^ “<5” is a peak detected under the lower limit of quantification (=5 μg/kg); ^(^^c)^ Concentration unit, mg/kg; ^(^^d)^ The acceptance limit with incorporated uncertainties in [ ]. Abbreviations: NIV, nivalenol; FUX, fusarenon-X; DON, deoxynivalenol, 3-ADON, 3-acetyl deoxynivalenol; 15-ADON, 15-acetyl deoxynivalenol; HT-2, HT-2 toxin; T-2, T-2 toxin; NEO, neosolaniol; DAS, diacetoxyscirpenol; FB_1_, fumonisin B_1_; FB_2_, fumonisin B_2_; FB_3_, fumonisin B_3_; FA_1_, fumonisin A_1_; FA_2_, fumonisin A_2_; FA_3_, fumonisin A_3_; ZEN, zearalenone.

**Table 3:** Concentration of *Fusarium* toxins in wheat samples.

Sample		Concentration of *Fusarium* Toxins [μg/kg]											
		Trichothecenes									Fumonisins		Zearalenone-group
		NIV	FUX	DON	3-ADON	15-ADON	HT-2	T-2	NEO	DAS	FB_1_	FB_2_	ZEN
Wheat	W-1	<5 ^(a)^		102	<5		<5	<5	<5	<5	<5		
	W-2	^(b)^		35.1				<5					
	W-3	<5	<5	19.0									
	W-4	<5		71.6			<5	<5					
	W-5	<5		405	21.3	16.9	10.1	<5					<5
	W-6			110			7.08	<5	<5				
	W-7	<5		352			<5	<5					
	W-8	<5	6.76	451	9.88		<5				<5		33.1
	W-9	<5		12.2									
	W-10	11.6	15.3	198		13.1	5.22	<5	<5	<5		<5	5.95
	W-11	6.99	25.5	271			<5						
	W-12	10.6	16.0	272			12.4	<5				<5	9.85
Barley	B-1			7.41									
	B-2	<5		6.56	34.1								
	B-3		<5										
	B-4		<5										
	B-5		<5										
	B-6	<5	<5	116		8.32	<5	<5					60.9
	B-7	<5											
	B-8			5.93		<5	<5	<5	<5	<5			
	B-9			77.7		<5	<5	<5		<5	0.43		

^(a)^ “<5” is a peak detected under the lower limit of quantification (=5 μg/kg); ^(b)^ The blank cells indicate that no peak was detected. Abbreviations: NIV, nivalenol; FUX, fusarenon-X; DON, deoxynivalenol, 3-ADON, 3-acetyl deoxynivalenol; 15-ADON, 15-acetyl deoxynivalenol; HT-2, HT-2 toxin; T-2, T-2 toxin; NEO, neosolaniol; DAS, diacetoxyscirpenol; FB_1_, fumonisin B_1_; FB_2_, fumonisin B_2_; ZEN, zearalenone.

## 3. Experimental Section

### 3.1. Samples, Chemicals, and Reagents

Thirty-four cereal samples, including 13 corn samples (grits and flour; C-1–13), 12 wheat samples (polished grains and flour, W-1–12), and 9 barley samples (polished grains and flour, B-1–9), were obtained from local supermarkets in Japan in 2015. Mycotoxin reference samples (DC-617, FC-443, ZC-327, MTC-9990, and MTC-9999E) obtained from Trilogy Analytical Laboratory (Washington, DC, USA) were used as reference corn samples naturally contaminated with mycotoxins.

MeOH (LC/MS grade), MeCN (analytical grade), acetic acid (guaranteed reagent grade), and ammonium acetate (analytical grade) were purchased from Kanto Chemical Co., Inc. (Tokyo, Japan). Water was purified using a Millipore (Molsheim, France) Milli-Q system. Q-sep Q 110 as a QuEChERS extraction kit was obtained from RESTEK (Bellefonte, PA, USA). MultiSep 229 Ochra as a multi-functional cartridge was obtained from Romer Labs (Bukit Merah, Singapore). A PTFE filter with mesh size of 0.20 μm was obtained from Advantec Toyo Kaisha, Ltd. (Tokyo, Japan). A Pierce LTQ Velos ESI Positive Ion Calibration Solution for positive mode calibration of Orbitrap MS was obtained from Thermo Fisher Scientific (Bremen, Germany).

The separation of 20 *Fusarium* toxins was compared using the following analytical columns: Mastro C18 (2.1 mm × 150 mm, 3 μm; Shimadzu GLC, Ltd., Tokyo, Japan), Mastro PFP (2.1 mm × 150 mm, 3 μm; Shimadzu GLC, Ltd. Tokyo, Japan), ACQUITY UPLC CSH Fluoro-Phenyl (2.1 mm × 150 mm, 1.7 μm; Waters, Milford, MA, USA), and Discovery HS F5 (2.1 mm × 150 mm, 3 μm; Supelco, Bellefonte, PA, USA).

The following standard solutions for each *Fusarium* toxin were used: NIV (100 μg/mL in MeCN), FUX (100 μg/mL in MeCN), DON (100 μg/mL in MeCN), 3-ADON (100 μg/mL in MeCN), 15-ADON (100 μg/mL in MeCN), HT-2 (100 μg/mL in MeCN), T-2 (100 μg/mL in MeCN), NEO (100 μg/mL in MeCN), and DAS (100 μg/mL in MeCN), from Wako Pure Chemical Industries, Ltd (Chuo-ku, Osako, Japan); and FB_1_ (50 μg/mL in MeCN/water (1:1 *v/v*)), FB_2_ (50 μg/mL in MeCN/water (1:1 *v/v*)), FB_3_ (50 μg/mL in MeCN/water (1:1 *v/v*)), ZEN (100 μg/mL in MeCN), α-ZEL (10 μg/mL in MeCN), β-ZEL (10 μg/mL in MeCN), α-ZAL (10 μg/mL in MeCN), and β-ZAL (10 μg/mL in MeCN) from Biopure Corp. (Cambridge, MA, USA). FA_1_, FA_2_, and FA_3_ were used by first acetylating the FB_1_, FB_2_, and FB_3_ standards, respectively [18,19].

### 3.2. Sample Preparation

Sample preparation was carried out as previously described [10,19]. Corn grits and polished grains were ground in a Labo Milser LM-PLUS (Iwatani, Tokyo, Japan) in advance. A 2.5 g sample was placed in a 50 mL polypropylene centrifuge tube and 20 mL of 2% acetic acid aqueous solution/MeCN (1:1, *v/v*) was added. The samples were mixed at 250 rpm using a shaker (SR-2 DS; Taitec Saitama, Japan) for 1 h. The contents of the Q-sep Q110 were then added to the centrifuge tube. The mixture was vortexed for 20 s and centrifuged at 1580 × *g* for 5 min. The supernatant (MeCN phase) was frozen at −30 °C for 1 h and was subsequently centrifuged at 1580 × *g* for 5 min. Next, 5 mL of the supernatant, 1 mL of water, and 60 μL of acetic acid were mixed, and the mixture was applied to the MultiStep 229 Ochra. The eluate (4 mL) was dried at 40 °C under a nitrogen stream and dissolved in 400 μL of 10 mM aqueous ammonium acetate/MeCN (85:15, *v/v*). Each sample was filtered using a 0.20 μm PTFE filter immediately prior to LC-Orbitrap MS analysis.

### 3.3. LC-Orbitrap MS Analysis

The LC-Orbitrap MS analysis was performed using an Ultimate 3000 system coupled to a Q-Exactive™ mass spectrometer (Thermo Fisher Scientific, Bremen, Germany). Xcalibur™ 2.2 software (Thermo Fisher Scientific, Bremen, Germany) was used to control the instruments and process the data.

LC was performed using a 10 mM aqueous solution of ammonium acetate as solvent A and 2% acetic acid in MeOH as solvent B. The gradient profile was 20% B (0 min), 40% B (1–2 min), 60% B (2 min), 70% B (9 min), 95% B (9–12 min), and 20% B (12–15 min). The flow rate was set to 0.3 mL/min and the column temperature was maintained at 40 °C. The chromatographic separation was carried out using a Mastro PFP column (2.1 mm × 150 mm, 3 μm) with an injection volume of 5 μL.

The Q-Exactive™ mass spectrometer was operated in positive mode with a heated electrospray ionization source (HESI-II) and a spray voltage of 3.00 kV. The capillary and heater temperatures were 350 °C and 300 °C, respectively. The sheath gas and the auxiliary gas flow rates were 40 and 10 arbitrary units, respectively. The correct mass calibration for analysis was performed following: (1) The calibration of the instrument was performed before each sequence using calibration solution; (2) The lock masses (*m/z* values of 188.98461 and 537.87906) were usually detected during the whole chromatographic run and were used for mass correction during the sequence. The precursor ion scan for determination was carried out in full MS mode at a resolution of 140,000 at an *m/z* value of 200 (3 scans/s), with an auto gain control (AGC) target of 3e6, a maximum injection time (IT) of 100 ms, and a scan range of 100–1000 *m/z*. Ammonium adduct ions [M+NH_4_]^+^ were selected for HT-2, T-2, NEO, and DAS; proton adduct ions [M+H]^+^ were selected for the other *Fusarium* toxins for quantification because of high sensitivity in positive mode. To judge the presence/absence of target toxins, the product ion scan was conducted in targeted MS^2^ mode using a resolution of 140,000 at an *m/z* value of 200, AGC target of 2e5, maximum IT of 200 ms, normalized collision energy (NCE) of 30 eV, stepped NCE of 50%, and scan range of 50–800 *m/z*. Table S2 shows the parameters for the 20 *Fusarium* toxins using the LC-Orbitrap MS measurements for quantification and certification.

### 3.4. Method Validation

The method was validated by evaluating the linearity, repeatability, and recovery. The coefficient of linearity was determined from calibration curves of the standard addition method constructed by plotting areas of prepared samples spiked with the 20 *Fusarium* toxins *versus* analyte concentrations. The concentrations of *Fusarium* toxin spiked to the test samples were 5, 10, 50, 100, 500, 1000, and 5000 μg/kg. Repeatability was assessed by calculating the relative standard deviation of five determinations in a single day. Recovery was assessed using samples spiked with each of the 20 *Fusarium* toxins. For repeatability and recovery, 100 μg/kg of each *Fusarium* toxin was spiked to the test samples at 100 μg/kg before the extraction process. The limits of quantification were defined as the lowest level of calibration curves (=5 μg/kg).

## 4. Conclusions

We have successfully developed a method for simultaneous analysis of 20 *Fusarium* toxins including 5 pairs of isomers in cereals. Good separation of these 20 *Fusarium* toxins using a Mastro PFP column and Orbitrap MS achieved to accurately detect these toxins in cereal matrix components with a mass error within ± 0.77 ppm. The validation of the developed method obtained good results. The result of the analysis of 34 commercially available cereals revealed that cereals have a risk of co-contamination with *Fusarium* toxins. Corn has an especially high risk of co-contamination with different groups of *Fusarium* toxins, and it tends to contaminate cereals at high concentrations. Thus, in the future, continuous control and monitoring of *Fusarium* toxins will be required to ensure food safety and protect economic investments, and here we report a method that will be useful in this task.

## Supplementary Materials

Supplementary materials can be accessed at: http://www.mdpi.com/2072-6651/7/5/1664/s1.
