# Supplementary material for: A Method for Simultaneous Determination of 20 Fusarium Toxins in Cereals by High-Resolution Liquid Chromatography-Orbitrap Mass Spectrometry with a Pentafluorophenyl Column
Source: Toxins (Basel). 2015 May 14;7(5):1664–82. doi: 10.3390/toxins7051664 (PMC4448167; doi:10.3390/toxins7051664)
Supplement: Supplementary file 1 [file toxins-07-01664-s001.pdf]

Supplementary Information

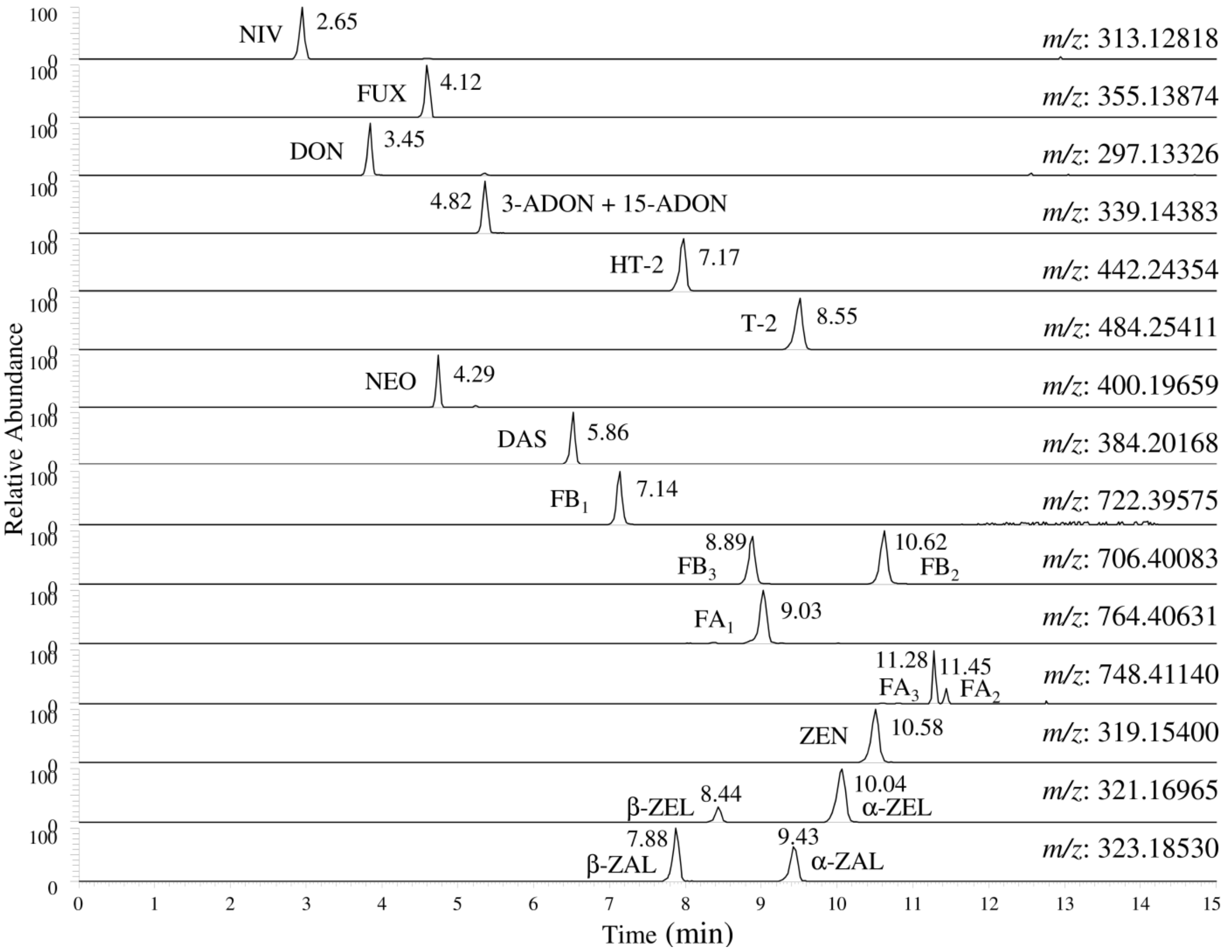

Figure S1. Chromatograms of 20 *Fusarium* toxins using a Mastro C18. The analytical sample was 200 µg/L of standards in neat solvent. Abbreviations: NIV, nivalenol; FUX, fusarenon-X; DON, deoxynivalenol, 3-ADON, 3-acetyl deoxynivalenol; 15-ADON, 15-acetyl deoxynivalenol; HT-2, HT-2 toxin; T-2, T-2 toxin; NEO, neosolaniol; DAS, diacetoxyscirpenol; FB_1, fumonisin B_1; FB_2, fumonisin B_2; FB_3, fumonisin B_3; FA_1, fumonisin A_1; FA_2, fumonisin A_2; FA_3, fumonisin A_3; ZEN, zearalenone; α-ZEL, α-zearalenol; β-ZEL, β-zearalenol; α-ZAL, α-zearalanol; and β-ZAL, β-zearalanol.

Table S1. The mass error of *Fusarium* toxins detected in the standards, a corn sample spiked with the 20 *Fusarium* toxins, and MTC-9999E.

***Fusarium* Toxins**	**Measured Ion**	**Fomula**	**Theoretical Mass (*m/z*)**	**Standard in Neat Solvent** [(a)]		**Corn sample Spiked with the 20 *Fusarium* Toxins** [(b)]		**MTC-9999E**	
				**Measured Mass (*m/z*)**	**Mass Error [ppm]**	**Measured Mass (*m/z*)**	**Mass Error [ppm]**	**Measured Mass (*m/z*)**	**Mass Error [ppm]**
NIV	$[M+H]^+$	$C_{15}H_{21}O_7$	313.12818	313.12815	−0.11	313.12799	−0.60		
FUX	$[M+H]^+$	$C_{17}H_{23}O_8$	355.13874	355.13868	−0.18	355.13894	−0.77		
DON	$[M+H]^+$	$C_{15}H_{21}O_6$	297.13326	297.13326	−0.01	297.13313	−0.44	297.13328	0.04
3-ADON	$[M+H]^+$	$C_{17}H_{23}O_7$	339.14383	339.14382	−0.04	339.14371	−0.36		
15-ADON	$[M+H]^+$	$C_{17}H_{23}O_7$	339.14383	339.14383	0.02	339.14382	−0.02		
HT-2	$[M+NH_4]^+$	$C_{22}H_{36}O_8N$	442.24354	442.24361	0.14	442.24357	0.07	442.24356	0.04
T-2	$[M+NH_4]^+$	$C_{24}H_{38}O_9N$	484.25411	484.25425	0.30	484.25405	−0.12	484.25423	0.25
NEO	$[M+NH_4]^+$	$C_{19}H_{30}O_8N$	400.19659	400.19666	0.15	400.19683	0.60		
DAS	$[M+NH_4]^+$	$C_{19}H_{30}O_7N$	384.20168	384.20164	−0.09	384.20145	−0.77		
FB_1	$[M+H]^+$	$C_{34}H_{60}O_{15}N$	722.39575	722.39562	−0.18	722.39551	−0.32	722.39559	−0.22
FB_2	$[M+H]^+$	$C_{34}H_{60}O_{14}N$	706.40083	706.40062	−0.30	706.40047	−0.51	706.40058	−0.36
FB_3	$[M+H]^+$	$C_{34}H_{60}O_{14}N$	706.40083	706.40066	−0.25	706.40064	−0.27	706.40070	−0.18
FA_1	$[M+H]^+$	$C_{36}H_{62}O_{16}N$	764.40631	764.40634	0.04	764.40631	−0.01		
FA_2	$[M+H]^+$	$C_{36}H_{62}O_{15}N$	748.41140	748.41151	0.15	748.41135	−0.07		
FA_3	$[M+H]^+$	$C_{36}H_{62}O_{15}N$	748.41140	748.41136	−0.05	748.41131	−0.12		
ZEN	$[M+H]^+$	$C_{18}H_{21}O_4$	319.15400	319.15397	−0.11	319.15384	−0.52	319.15399	−0.02
α-ZEL	$[M+H]^+$	$C_{16}H_{25}O_5$	321.16965	321.16971	0.17	321.16954	−0.34		
β-ZEL	$[M+H]^+$	$C_{16}H_{25}O_5$	321.16965	321.16965	−0.01	321.16962	−0.10		
α-ZAL	$[M+H]^+$	$C_{18}H_{27}O_5$	323.18530	323.18543	−0.13	323.18551	0.63		
β-ZAL	$[M+H]^+$	$C_{18}H_{27}O_5$	323.18530	323.18532	0.07	323.18542	0.38		

[(a)] The analytical sample was 200 µg/L of standards in neat solvent; [(b)] The analytical sample was a corn spiked with 100 µg/kg of standards. Abbreviations: NIV, nivalenol; FUX, fusarenon-X; DON, deoxynivalenol, 3-ADON, 3-acetyl deoxynivalenol; 15-ADON, 15-acetyl deoxynivalenol; HT-2, HT-2 toxin; T-2, T-2 toxin; NEO, neosolaniol; DAS, diacetoxyscirpenol; FB_1, fumonisin B_1; FB_2, fumonisin B_2; FB_3, fumonisin B_3; FA_1, fumonisin A_1; FA_2, fumonisin A_2; FA_3, fumonisin A_3; ZEN, zearalenone; α-ZEL, α-zearalenol; β-ZEL, β-zearalenol; α-ZAL, α-zearalanol; and β-ZAL, β-zearalanol.

Table S2. The parameters of the 20 *Fusarium* toxins by LC-Orbitrap MS measurement.

***Fusarium* Toxin**	**Measured Ion**	**Measured Precursor Ion for Quantification (*m/z*)**	**Measured Product Ions for Certification (*m/z*)**	**Retention Time [min]**
NIV	$[M+H]^+$	313.12818	137.05971/295.11761	2.99
FUX	$[M+H]^+$	355.13874	137.05971/247.09649	4.55
DON	$[M+H]^+$	297.13326	203.10666/249.11214	3.85
3-ADON	$[M+H]^+$	339.14383	203.10666/231.10157	5.50
15-ADON	$[M+H]^+$	339.14383	137.05971/321.13326	5.35
HT-2	$[M+NH_4]^+$	442.24354	215.10666/263.12779	7.88
T-2	$[M+NH_4]^+$	484.25411	185.09609/215.10666	9.88
NEO	$[M+NH_4]^+$	400.19659	215.10666/305.13835	4.64
DAS	$[M+NH_4]^+$	384.20168	247.13287/307.15400	6.49
FB_1	$[M+H]^+$	722.39575	334.31044/352.32101	8.73
FB_2	$[M+H]^+$	706.40083	318.31553/336.32609	11.57
FB_3	$[M+H]^+$	706.40083	318.31553/336.32609	10.67
FA_1	$[M+H]^+$	764.40631	728.38518/746.39575	8.21
FA_2	$[M+H]^+$	748.41140	318.31553/730.40083	11.11
FA_3	$[M+H]^+$	748.41140	336.32609/378.33666	10.42
ZEN	$[M+H]^+$	319.15400	187.07536/283.13287	11.95
α-ZEL	$[M+H]^+$	321.16965	189.09101/303.15909	11.78
β-ZEL	$[M+H]^+$	321.16965	285.14852/303.15909	10.77
α-ZAL	$[M+H]^+$	323.18530	123.04406/305.17474	11.44
β-ZAL	$[M+H]^+$	323.18530	189.09101/305.17474	9.61

Abbreviations: NIV, nivalenol; FUX, fusarenon-X; DON, deoxynivalenol, 3-ADON, 3-acetyl deoxynivalenol; 15-ADON, 15-acetyl deoxynivalenol; HT-2, HT-2 toxin; T-2, T-2 toxin; NEO, neosolaniol; DAS, diacetoxyscirpenol; FB_1, fumonisin B_1; FB_2, fumonisin B_2; FB_3, fumonisin B_3; FA_1, fumonisin A_1; FA_2, fumonisin A_2; FA_3, fumonisin A_3; ZEN, zearalenone; α-ZEL, α-zearalenol; β-ZEL, β-zearalenol; α-ZAL, α-zearalanol; and β-ZAL, β-zearalanol.

© 2015 by the authors; licensee MDPI, Basel, Switzerland. This article is an open access article distributed under the terms and conditions of the Creative Commons Attribution license (http://creativecommons.org/licenses/by/4.0/).
